# While You Were Sleepwalking: Science and Neurobiology of Sleep Disorders & the Enigma of Legal Responsibility of Violence During Parasomnia

**DOI:** 10.1007/s12152-015-9229-4

**Published:** 2015-04-24

**Authors:** Shreeya Popat, William Winslade

**Affiliations:** The University of Texas Southwestern Medical School, Dallas, TX USA; Plan II Honors Program, The University of Texas, Austin, TX USA; The Institute for the Medical Humanities, The University of Texas Medical Branch, Galveston, TX USA

**Keywords:** Somnambulism, Violence, Sleep arousal disorders, Parasomnias, Crime

## Abstract

In terms of medical science and legal responsibility, the sleep disorder category of parasomnias, chiefly REM sleep behavior disorder and somnambulism, pose an enigmatic dilemma. During an episode of parasomnia, individuals are neither awake nor aware, but their actions appear conscious. As these actions move beyond the innocuous, such as eating and blurting out embarrassing information, and enter the realm of rape and homicide, their degree of importance and relevance increases exponentially. Parasomnias that result in illegal activity, particularly violence, are puzzling phenomena for medicine and the law. Via a review of the pertinent medical literature, a general overview of the current scientific knowledge of parasomnias will be provided. Though this knowledge is far from complete, it can provide some neurobiological information about the nature of parasomnia, including conclusions about a sleepwalker’s level of intention as well as factors that predispose one to such episodes. Although a parasomniac’s complete lack of consciousness warrants acquittal from criminal liability, it does not exclude responsibility for subjecting oneself to exacerbating factors that result in these violent parasomnias. Individuals should be held accountable if they could be expected to control these factors. In addition, they should undergo appropriate treatment and management in order to prevent future parasomnia behaviors. Establishing a legal defense for parasomnia will prove difficult due to the strong potential for malingering, so specific criteria will be outlined in order to distinguish between true and fraudulent claims of crimes committed during parasomniac states.

## Introduction

Several mornings in a row, a college acquaintance of mine woke puzzled by the fact that he kept finding food in his bed. Over the last month, his wrestling coach had been pressuring him to gain weight in order to move up a weight class, and he became so stressed about it that he started to sleepwalk. He would get out of bed, walk to his kitchen, and get food from his fridge and pantry. Then, he would carry it back to his bed and eat until his episode of “sleep-eating” ended. In the morning, he would wake up surrounded by food.

Such night wanderings are pervasive. They appear in friends’ anecdotes, in cartoons, on television, etc. Sleepwalking has become such a widely known phenomenon that its mention quickly conjures a characteristic image of a person meandering with arms outstretched and eyes glazed over. As strange but harmless ventures out of bed, they provide entertaining stories to share and unique phenomena to explore. However, when these episodes transition from sleepwalking to a sort of “sleep-acting,” these actions become problematic.

During the night of May 23, 1987, Kenneth Parks rose from his bed and wandered out of his house and into his car, driving 14 miles to the home of his mother- and father-in-law in the neighboring town. Upon arriving at their home, he took a tire iron from the car and entered the house. He then beat his mother-in-law to death and choked his father-in-law. Now a blood-spattered mess covered in cuts and bruises, he drove to a nearby police station. In a confused manner, he told the police that he “thought” he killed someone. Though still confused, he identified his in-laws, murmuring that it was “all his fault.” Eventually, Parks was tried for one charge of murder and one charge of attempted murder. His defense claimed that, during the entire episode, he was sleepwalking [1].

The idea that a person can execute a coordinated act of violence in their sleep opposes the stereotypical perception that sleepwalkers are in a mindless daze, performing actions over which they have no control or awareness [2]. In this manner, they present a troublesome enigma. While these cases do not conform to the stereotype, whether they overthrow it or are just disingenuous is unclear. Also, they bring a medical phenomenon into the realm of legal responsibility. Assuming that sleepwalking can actually result in violence, the legal system must figure out how to approach these unique cases. To address these concerns, current medical knowledge must be united with legal standards.

## The Stages of Sleep

To categorize the different stages of sleep, three standard laboratory measures are used. The first, an electroencephalogram (EEG), records brain activity in the form of waves. The second, an electrooculogram (EOG), notes eye movements. Finally, an electromyogram (EMG) measures muscle activity via a sensor placed on the chin (or alternatively on the arm or leg).

Sleep is divided into two parts: rapid eye movement (REM) sleep and non-rapid eye movement (NREM) sleep. When one first falls asleep, one usually enters NREM sleep, which is divided into four stages. During the first stage, thoughts start to drift, and the ability to react to external stimuli begins to decrease [3, 4]. Muscle activity begins to slow [3, 4]. As one moves through the stages of NREM sleep, EEG waves grow in amplitude and decrease in frequency – they become bigger and slower. The “deep” stages of sleep are NREM stage 3 and 4. Waking someone during these stages of sleep often proves quite difficult, and the person awakens feeling groggy and disoriented [4].

After the four stages of NREM sleep, one moves into REM sleep, where dream mentation occurs and manifests as some cerebral activity on the EEG [3]. During this stage, the EOG shows rapid eye movements in random directions, and the EMG indicates a complete lack of muscle tone [4]. This muscle atonia prevents the individual from acting out their dreams. If one attempts to wake a person in REM sleep, it is usually effortless, and the person wakes up with little disorientation [3]. Each complete sleep cycle (with both NREM and REM sleep) lasts approximately 90 minutes in adults [4]. As the night progresses, the time spent in stages 3 and 4 of NREM sleep decreases while the time spent in REM sleep increases [5].

## Clinical Aspects of Sleep Disorders

Sleep disorders are generally divided into two major categories. Dyssomnias refers to the group of sleep disorders that manifests as “excessive sleepiness or difficulty in initiating or maintaining sleep” [6, 7]. This includes insomnia, narcolepsy, and circadian rhythm disorders (such as jet lag and shift work disorder) [8]. Dyssomnias rarely become violent in a manner in which they could be mistaken for awake and aware actions. Parasomnias, the second category of sleep disorders, occur during sleep and are marked by significant skeletal muscle activity [6, 7]. Simply put, parasomnias are disorders that result in physical actions that are completely uncharacteristic of sleep [9]. Many disorders within the category of parasomnias are beyond the scope of this discussion as they, like dyssomnias, rarely manifest in violence. Two disorders are of particular interest in this discussion: somnambulism and REM sleep behavior disorder (RBD) [10]. Though they can both result in seemingly coordinated violent actions, the two disorders are actually quite different.

Somnambulism, colloquially known as sleepwalking, appears as episodes of motor activity ranging from walking to driving and even violence among a variety of other inappropriate (for sleep, that is) actions. It occurs during the deepest stages of NREM sleep, stages 3 and 4. Therefore, somnambulism is more likely to occur in the earlier part of the night when these stages predominate. It is more prevalent in males between 7 and 15 years of age, though somnambulism that results in violence usually occurs in a slightly older age group. The episodes typically last from a few minutes to an hour and can occur from once a month to multiple nights per week. Attempts to wake individuals usually remain fruitless and sometimes even elicit violent responses, such as aggressive and spastic limb movements [11, 12]. During the episode, the individual appears awake but is unresponsive [11]. Sensory perceptions are virtually switched off, so while the person can often navigate around objects due to habit and simple stumbling, he does not fully perceive any sight, smell, sound, or even pain [13]. The individual’s eyes are usually directed upward and inward, resulting in a vacant expression [12, 13]. In addition, memories of the incident do not carry into the waking state [11]. If awakened during a sleepwalking episode, the individual usually remains confused and disoriented for a period of time [11].

Recently, scholars have begun viewing somnambulism as a new type of state dissociation. In this theory, the states of wakefulness and sleep are not mutually exclusive and can mix or oscillate rapidly [14]. A combination of complex multifaceted mechanisms contributes to each state. The human body seems to have constructed a reliable way to ensure that all of these mechanisms usually synchronize. During state dissociation episodes, a majority of the mechanisms line up for one particular state. In the case of somnambulism, the body’s physiological mechanisms prepare to enter the deep stages of NREM sleep [14]. However, some important mechanisms do not occur. In somnambulism, significant motor activity (an aspect of wakefulness) remains [14]. EEGs depict a mix of the large amplitude, low frequency waves characteristic of stage 3 and 4 NREM sleep along with the low amplitude, high frequency waves found in awake states [15]. Therefore, according to state dissociation theory, somnambulism is an amalgam of NREM sleep and wakefulness.

In contrast, REM sleep behavior disorder (RBD) occurs during the REM stage of sleep. To recap, REM sleep is marked by elaborate dreams as well as muscle atonia that prevents individuals from acting out their dreams [16]. In RBD, this muscle atonia is disabled intermittently [16]. In terms of state dissociation theory, RBD is considered a combination of REM sleep and wakefulness (due to the presence of muscle tone) [14]. This results in a variety of behaviors that can range from talking or screaming to thrashing one’s limbs about in bed and even more seemingly coordinated violence. However, individuals with RBD tend to awaken as their feet hit the floor or soon after, so violent actions usually target the bed partner or those nearby [14]. Because RBD is essentially acting out one’s dreams, the individual will remember bits and pieces of the events from the episode in the form of a vivid but disconnected dream [17]. Furthermore, since one can awaken more easily from the lighter REM sleep, the individual with RBD will not appear as disoriented afterwards [17]. Finally, RBD usually occurs in the later parts of sleep in the early morning, when more time is spent in REM sleep [18].

Recently, a strong connection between RBD and a group of degenerative neurological diseases, the synucleinopathies, has begun to emerge [7, 19–22]. This group includes Parkinson’s disease, dementia with Lewy body disease, and multiple system atrophy [18]. In one series of cases, over 2/3 of patients initially diagnosed with RBD eventually developed symptoms of one of the synucleinopathies [18, 21]. In this group of neurological disorders, an aggregate fibrillar structure of a protein called alpha-synuclein forms characteristic pathologic lesions called Lewy bodies [23]. Unfortunately, the physiological role of alpha-synuclein is still unknown.

At this point, let us revisit the case of Kenneth Parks, the man who killed his mother-in-law and severely injured his father-in-law and claimed he was sleepwalking. Examining Parks’ account of his episode and the nature of the actions, it is arguable that Parks was most likely in an episode of somnambulism rather than RBD. First, his episode was quite lengthy, allowing him to drive 14 miles. Furthermore, Parks had absolutely no memory of the situation, not even a minor recollection of any fragmented dreams, which would occur with RBD. Finally, Parks still appeared out of sorts when he arrived at the police station after the incident [1].

## Neurobiology of Parasomnias and Correlates with Legal Responsibility

Alpha motor neurons control skeletal muscle fibers, which are responsible for the majority of body movements. During sleep, the input signals to these neurons change. Excitatory impulses, which depolarize these neurons, decrease. Inhibitory signals, which hyperpolarize the alpha motor neurons, increase. To reach the threshold potential in a hyperpolarized cell requires a significantly greater net depolarization, i.e., a much larger excitatory impulse, than achieving the threshold from resting potential. Therefore, transmitting an action potential to skeletal muscle is much less likely. During REM sleep, the alpha motor neuron membranes are significantly more hyperpolarized, making it even more difficult for impulses to fire and cause movement [24].

Scientists initially believed that motor activity relied on the cerebrum, and “lower brain structures” only sustained “vegetative” activities like breathing [25]. However, new discoveries have found that “extremely complex emotional and motor behaviors can originate from [the brain stem and cerebellum] without involvement of ‘higher’ neural structures” [25]. Regions of the brainstem and the cerebellum have been found to contribute to the activity of motor neurons [24, 26]. Lesion studies have shown that cell damage in specific areas in the brainstem and cerebellum are connected with cases of retention of muscle tone, i.e., suspension of muscle atonia, during REM sleep and with seemingly conscious wandering and violent movements [12, 19].

The cerebrum and cerebral cortex control and regulate actions, rather than initiate them as was previously thought [25]. In humans, these structures are significantly more developed than other animals [25]. They contribute to the “higher” intellectual abilities we typically attribute to people, such as judgment, intention, reasoning, etc. [27]. Claudio Bassetti of the University of Zurich has examined the brains of sleepwalkers [28]. Using single photon emission computerized tomography (SPECT), he has noted that the cerebellum and brainstem are active while the cerebrum and cerebral cortex show little activity in sleepwalkers’ brains [28]. Coordination and integration of information between different parts of the cerebral cortex is considered an important precursor for consciousness [14]. Therefore, while asleep, an individual essentially acts without any consciousness. Parts of the cerebrum also contribute to sensory processing and the formation of new memories, which explains why individuals do not remember episodes of parasomnia.

The absence of consciousness during parasomnias can be verified from more indirect evidence as well. Sensory perception, voluntary control of motor activity, and the ability to interact with and experience the world are considered important aspects of consciousness. During episodes of parasomnia, individuals do not have any sensory perception, memory formation, or responsiveness towards the environment – they have absolutely no mental interaction with the outside world. In other words, they are completely unaware during an episode of parasomnia. Compare this to patients in a vegetative state. These patients go through typical sleep and wake cycles but are unconscious and unresponsive. In this sense, they are often referred to as “awake but not aware.” Individuals in parasomnia episodes are neither awake nor aware. Since we consider individuals in a vegetative state unconscious, we would consider individuals in an episode of parasomnia to be even less conscious, if that is even possible.

To revisit the story of Kenneth Parks, if he was truly sleepwalking during the attack on his in-laws, he had no conscious intention to harm them. This point actually came up during his trial. His relationship with his in-laws was thoroughly investigated; his wife even testified that he had no motive to harm them [1]. Even though Parks had been accused of embezzlement and was going to be taken to court in the near future, his mother- and father-in-law had always been supportive [1]. The inability to form conscious intentions is a prominent characteristic of parasomnias that has significant legal ramifications.

## Actus Reus and Mens Rea: Freedom from Responsibility in Legal Theory

To understand how the law deals with violence committed during parasomnia, a preliminary review of legal theory is required. Common law legal systems, found in Great Britain and most of its former colonies, discuss two components to every crime: *actus reus*, which is the criminal act itself, and *mens rea*, which is the criminal intent [29]. These components are derived from the Latin phrase *Actus non facit reum nisi mens sit rea*, which means “the deed does not make a man guilty unless his mind is guilty” [30]. If one of these components is absent, the defendant is acquitted. In the cases in question, *mens rea* is absent. Moreso, as mentioned in the previous section, the individual is neither awake, aware, nor able to make a decision to not act – such actions are legally termed “automatisms.” Several different definitions have arisen for this term. Some focus on the lack of awareness: the psychiatrists Hinsie and Shatzky assert that it is “a condition in which activity is carried out without conscious knowledge on the part of the subject” [30]. Other definitions focus on the lack of decision-making power, like Peter Fenwick who theorizes that “an automatism is an involuntary piece of behavior over which an individual has no control” [30]. Under either definition, parasomnias are clearly automatisms. Automatisms usually lead to legal acquittal due to the lack of *mens rea*.

Currently, automatisms are commonly divided into two categories: sane and insane automatisms. This division has been revisited most frequently in British law, where such cases have appeared for many decades. Sane automatisms result from an external factor, such as a blow to the head, and result in complete acquittal. Insane automatisms are due to some sort of internal factor like a brain tumor or a psychological disorder, referred to as “diseases of the mind;” they traditionally result in compulsory confinement in a psychiatric facility.

These categories have caused significant confusion in cases where medicine and law intersect. For instance, in the 1955 case *R v. Charlson*, a British court classified an act that resulted from a cerebral tumor as a sane automatism despite a cerebral tumor appearing an internal causal factor [31]. In this case, the judge focused on the connotations of “sanity” and “insanity:” he acknowledged that a cerebral tumor was not analogous to psychological insanity and did not warrant confinement in a mental asylum. However, two years later in *R v. Kemp*, “an automatic act committed in a confusional state due to arteriosclerosis” was labeled an insane automatism [32]. The judge in *Kemp* focused on the fact that the cause was internal and affected the defendant’s reasoning ability, not on how it fit the connotation of insanity or on the associated penal consequences. Then, in 1963, the case *Bratty v. Attorney*-*General for Northern Ireland* addressed the issue of violence during epileptic episodes [33]. The presiding judge Lord Denning opined that “any mental disorder which has manifested itself in violence and is prone to recur is a disease of the mind,” i.e., an insane automatism [33]. While epilepsy appears to fit this statement, Lord Denning excluded it from this category, as he chose to focus instead on the penal consequences [33]. He stated that epilepsy “is the sort of disease for which a person should be detained in hospital rather than be given an unqualified acquittal” or be admitted to a psychiatric facility [33]. Though this comment could be extrapolated to parasomnias, British courts have continued to classify violence during parasomnias as insane automatisms. While these judgments were of different courts throughout the United Kingdom, it is evident that these categories have caused conflicting legal decisions and failed to establish a clear precedent [30]. This is particularly concerning in common law tradition, which is largely based on precedent.

In fact, the distinction between sane and insane automatisms presents a dilemma for discussions of violence committed during episodes of parasomnia. Almost all scholars consider parasomnias an effect of some sort of neurological sleep disturbance. With this internal type of cause, violent actions performed during parasomnias seem to fall into the category of insane automatisms. However, it hardly seems appropriate to place individuals who sleepwalk in mental institutions – they are far from mentally ill. Still, it is inappropriate to let parasomniacs walk away from such violent actions free of any legal responsibility and with no further requirements to take proper steps to manage their parasomnias. Therefore, classification as a sane automatism seems insufficient as well. Overall, the implication of the sanity/insanity distinction is one that makes little sense with regard to parasomnias and many other medical issues as well.

## A Novel Perspective on Automatisms

Before considering proposals for a better system for the legal categorization of parasomnias, it is important to note that parasomnias usually surface and result in violence due to exacerbating factors. These can include family history, drug/alcohol abuse, stress, and/or sleep deprivation, among others. Since many of these factors are external, they would be categorized as sane automatisms. However, many idiopathic cases of parasomnia as well as those associated with family history would be categorized as insane automatisms. Clearly, the current endeavor to label violence during parasomnia as a sane or insane automatism is counterintuitive. It is better to appreciate that violence during parasomnias manifests in different ways and is evoked by different factors in different individuals. In concordance, there should be different levels of legal responsibility and appropriate legal consequences. The unclear dichotomy between sane and insane automatisms cannot be applied easily and sensibly.

Instead, consider a gradient based upon a notion of a reasonable degree of control. It would lead to more fruitful prescription of legal responsibility and consequences. On one extreme would be automatisms caused by factors within one’s control. Individuals with such automatisms would be held legally responsible for their actions. Aristotle alludes to this concept with an example in his work *Ethics*: even though a drunken individual does not act voluntarily or with full capacity while he is intoxicated, he acts voluntarily and with mental capacity when choosing to become intoxicated and should therefore be held accountable for doing so [34]. An individual whose parasomnia episodes are triggered by excessive alcohol consumption or other factors within their control mirrors this example. Under current jurisprudence, such a case would be classified as a sane automatism since alcohol consumption is an external factor, and the parasomniac individual would be acquitted. By contrast, in the new schema, the actions of the parasomniac individual would be classified as automatisms triggered by factors within one’s control, and the individual would be held legally responsible for his actions.

Though this new manner of classification is not foolproof, it is clearly an improvement from the sane vs. insane automatism schema. To determine how to classify an automatism, the operative question would be “Could the individual, at the time that the instigating factor(s) came into play, be reasonably expected to act differently so as to change the outcome?” This question gauges the level of control and decision-making ability involved in the circumstances that put one in a vulnerable position for violent parasomnias. Consider this question in one of the previous examples. For the individual who sleepwalks due to a family history, the answer to this question would be no. It is important to note that he could still attempt to actively manage his sleepwalking and prevent putting himself in situations that further exacerbate his tendency to sleepwalk, so a family history should not automatically warrant release from responsibility.

This new schema provides a meaningful division with regard to the question of legal responsibility. People who exhibit automatistic behavior due to factors out of their control should not be held responsible for their actions while people who perform automatisms due to factors within their control should be held accountable. There is some middle ground between these two extremes that can be left to the interpretation of the judge and jury.

There is also a more natural division in terms of legal consequences. Since people who exhibit automatistic behavior due to factors out of their control cannot reasonably be held responsible for their actions, they should be acquitted for a criminal offense committed while in such an automatistic state. Still, while they are not guilty of the crime in itself, they should be held accountable for seeking appropriate treatment or intervention options to manage or eliminate their particular sleep disorder. Consider this qualification in the context of cases in which even a moderate amount of a particular prescription medication or alcohol can evoke parasomnias. For instance, think of a family that is particularly sensitive to tequila. If any of the individuals in this family drink even a small amount of tequila, they will sleepwalk and, during these sleepwalking episodes, attempt to engage in dangerous behaviors such as attempting to drive. Consider this in the context of the key question: “Could the individual, at the time that the instigating factor(s) came into play, be reasonably expected to act differently so as to change the outcome?” This family, because they are aware of the likelihood of their adverse reaction to tequila, could be reasonably expected to act differently so that they do not sleepwalk and put themselves and others in harm’s way. However, consider this paradigm in the context of an individual who is not aware of his heightened sensitivity to a particular drug or alcohol. For example, the medication zolpidem (Ambien) has been shown to produce parasomnias in some individuals even when taken as directed [35–37]. If an individual took the medication for the first time in the manner in which he was instructed and had no reason to suspect that this could cause him to act in a dangerous or uncharacteristic way, any resulting automatisms are beyond his control. No one can reasonably expect these individuals to not take a medication that a doctor tells them will assuage their complaints.

Under the proposed system of classification, an initial pardon should be applied for these scenarios. Assuming the amount of drug/alcohol consumption was reasonable and that the individual had no reason to suspect an adverse reaction from consumption, his first incident would be excused, as he cannot reasonably be expected to act any other way. However, once he realizes the substance’s effects on him, he should know to avoid the drug or alcohol that evokes parasomnias and subsequent violence. If an additional offense occurs after the individual can be reasonably expected to be aware of his sensitivity, then he would be held responsible. In these contrasting scenarios, the question “Could the individual, at the time that the instigating factor(s) came into play, be reasonably expected to act differently so as to change the outcome?” provides an adequate rule of thumb.

On the other hand, people who commit violent automatisms due to factors that are within their control can be held responsible for their actions and should be tried in a court of law. However, since the automatism had no *mens rea*, the automatistic act itself is not considered the crime. Instead, they should be held accountable for putting themselves in the situation where they are vulnerable to violent parasomnias. In other words, they should be held responsible for the factor over which they had control (such as drug abuse, alcohol abuse, etc.) and tried accordingly for their recklessness and/or failure to take appropriate precautions. This can be compared to the recent legal cases in which sleep-deprived drivers have been held accountable for deaths that result from them falling asleep behind the wheel – usually, it is a charge of manslaughter. In these cases, a stipulation is usually made that the sleep deprivation has to result from some sort of recklessness or gross negligence on the driver’s part. For example, think of a college student who pulls an all-nighter and then gets in a car and finds himself falling asleep at the wheel. This student had ample opportunity to choose not to pull the all-nighter or choose not to drive, especially when he realized he was falling asleep while driving. He would be held responsible for any resulting harm. Sleep deprivation is also an exacerbating factor for parasomnias, so this same example can be extrapolated to violent acts performed during episodes of parasomnia, especially if violent automatisms were a foreseeable consequence of the sleep deprivation.

Applying this new classification system to the case of Kenneth Parks proves quite interesting. During his trial, his defense presented Parks’ history of sleepwalking since childhood and proclivity for long, elaborate episodes of sleepwalking when exposed to various exacerbating factors, especially mental stress [1]. At the time of the incident, Parks was incredibly anxious due to his upcoming embezzlement trial [1]. From the numerous arguments of his defense to support a defense of sleepwalking, it seemed evident that Park should have been reasonably aware of his tendency to sleepwalk, his sensitivity to stressors, and his likelihood of engaging in dangerous activities like driving while sleepwalking. Therefore, it can be considered negligent for him not to have sought medical care and taken appropriate precautions to, if not prevent sleepwalking, at least ensure his and others’ safety during these episodes. In the context of the significant mental stress from his embezzlement trial, it can even be considered reckless on his part. Therefore, Parks’ violence would fall into the category of automatisms due to factors within the defendant’s control.

The entire discussion of Parks’ case up to this point has functioned on the assumption that his plea of parasomnia was an honest one. It is important to consider the possibility that Parks was lying, and it was not a true case of somnambulism. During his trial, this was hotly debated – each side grew fierce in their arguments, and both made plausible claims in favor of their opinions [1].

## Qualifications for a Plea of Parasomnia

When establishing a legal defense for violence committed during parasomnia, it is important to consider the potential abuse of such a defense. A stringent and unbiased method for filtering through these pleas can combat many cases of malingering. Unfortunately, there is a limit to the level of rigidity possible in these criteria for several reasons. First, current knowledge on sleep disorders is still somewhat limited, and future data and research could greatly enhance our understanding. Second, parasomnias vary from individual to individual, so creating a universally applicable set of criteria is extremely difficult. The best compromise is a set of basic, flexible guidelines that suggest what a jury should consider when distinguishing a true case of parasomnia from a fake one:

1. Results of clinical polysomnographic tests
  The most definitive way to diagnose a sleep disorder is an in-depth polysomnography test [38]. Conducted over several nights, these tests consist of an EEG, EOG, and EMG to measure any abnormalities in brain, muscle, and/or eye activity. Sleep experts with many years of experience should interpret the results of these tests. After several thorough overnight polysomnographic examinations, they can postulate about how likely it is that a particular person has a sleep disorder and introduce these possibilities to the court [38].
2. Contribution of an unbiased medical practitioner
  In addition to an expert’s interpretation of the polysomnography results, it is imperative to involve an experienced medical practitioner, particularly one knowledgeable in forensic aspects of parasomnias, in the trial itself. First, they can educate the judge and jury about the legitimate possibility of violence during an episode of parasomnia. Second, they can guide the jury through the criteria for diagnosis and confirmation of a parasomnia, explaining which aspects of the episode in question indicate a true parasomnia and which seem to indicate that one did not occur.
  Within the current adversarial legal system, the prosecution and the defense involve their own expert witnesses and reimburse these experts of their own accord. Unfortunately, in the past, this has led to subtle biases, devolved into character attacks, and overall confused the jury, especially in heated cases such as those of Kenneth Parks [1]. Efforts should be made to prevent this, possibly by eliminating the partisan nature of expert testimonies and introducing court-appointed expert witnesses that present both supporting and opposing evidence. However, such a discussion is beyond the scope of this work.
3. Time of the night
  As mentioned, cases of violence during somnambulism are more likely to occur at earlier times of the night, within 3 to 4 hours of the individual falling asleep. On the other hand, RBD typically occurs during the later part of the night, closer to the morning. This is simply a correlation between the general population’s physiological sleep cycle and the pathophysiology of RBD and somnambulism. It can only serve to support existing reasoning for a diagnosis. Alone, the time of the night during which the episode of violent parasomnia occurs is a weak support for a claim of parasomnia. Still, it can strengthen claims, as it is a consistent observation.
4. Exacerbating factors, i.e., stress, sleep deprivation, alcohol, fever, drugs, and/or breathing disorders
  As stated previously, most sleep disorders surface due to an exacerbating factor. Because these factors can vary widely from person to person, an expert opinion and clinical polysomnography testing are invaluable in highlighting the presence and likelihood of an exacerbating factor specific to the defendant. The factors listed above are the most common [39]. They each strain the body in some manner, which manifests during sleep as sleep disturbances [39]. Breathing disorders, such as obstructive sleep apnea, sleep-disordered breathing, and upper airway resistance syndrome, cause hypercapnia and hypoxia which then arouse the body in an attempt to trigger muscles involved in breathing. If motor control neurons that contribute to walking and other motor functions are also activated, this can cause parasomnia.
  Though large-scale studies on alcohol consumption resulting in parasomnias have not been conducted, many case reports describe such examples [40]. Among scholars, this has led to the acceptance of alcohol as a contributing factor towards parasomnias, especially violent ones. In addition, studies show that during the first part of sleep, alcohol significantly increases the amount of NREM deep sleep [39]. This would increase time and opportunity for an episode of somnambulism to occur during the first part of sleep, which is when it is already most likely to occur (as discussed previously). During the second half of sleep periods, alcohol increases time spent in REM sleep and also makes sleep lighter and more easily disrupted [39]. As discussed earlier, RBD is more likely to occur during the latter half of a sleep period, and these changes would further increase opportunity for an episode.
  Finally, different drugs have also been linked to sleepwalking. To differentiate between appropriate drug use and abuse and determine the possibility that a particular drug impacted sleep, a medical expert witness should be involved since a thorough list of potential drugs is continuously evolving. The medical expert can present up-to-date pharmaceutical research and case reports and explain how they relate to the case.
5. Family history
  Somnambulism is typically associated with a strong family history [39]. The “presence of sleepwalking or a related disorder in a first-degree relative increases the chances of developing this disorder by a factor of 10” [39]. Also, in individual case studies of sleepwalkers, a family history of sleepwalking is usually present. A recently published study identified the HLA gene DQB1, which is present in 35 % of sleepwalkers (as compared with 13.3 % of non-sleepwalkers) [39]. This is an interesting diagnostic finding though not complete, for 65 % of sleepwalkers still lack the gene.
  Few studies comment on a hereditary correlation in RBD. However, since Parkinson’s, Lewy Body disease, and the other alpha-synucleinopathies often manifest over several generations, it is likely that RBD could as well [21]. For this reason, documented or witnessed accounts of first degree relatives experiencing associated sleep disturbances can support a case of alleged parasomnia, either RBD or somnambulism [22].
6. Witness accounts of the characteristics of the accused
  People who observed the defendant during the episode can attest to how the defendant appeared, which is important for confirmation of parasomnia. A witness can attest to how lucid and responsive the defendant appeared as well as how he performed the actions – in a coordinated and calculated way or in more haphazard manner. They could also provide information about other aspects of the episode, such as if the defendant sought out the victim and how the defendant appeared upon waking: if he appeared surprised and astonished at where he found himself, if he seemed disoriented, if he tried to cover up his actions, etc. [25]. The description can then be compared with the expected demeanor of a parasomnia, as described by the medical expert witness. Since the defendant, in a case of true parasomnia, would not remember the episode, a secondary opinion is the closest account a jury can receive.
7. Intentionality of the violence
  In a 2007 series, Mark Pressman examined 32 cases of violence during somnambulism in medical and legal literature. In a majority of these cases, he noted that closeness and proximity played a prominent role [41]. Individuals typically did not seek out the targets of their violence. Rather, they came across them circumstantially, or the targets approached and/or provoked them [42]. Though this case series includes a small and nonrandomized sample, proximity and provocation still appear to be significant triggers of violent behaviors during somnambulism [41]. This eliminates the notion of motivation on the sleepwalker’s part. Usually, victims of this violence are not even people that the sleepwalker knows or towards whom he or she feels begrudged, just people whom circumstance brought into the proximity of the sleepwalker. It is important to note that Kenneth Parks, who drove 14 miles and attacked his mother- and father-in-law, is an exception to this pattern. His defense successfully argued that he routinely drove the 14 mile route to the home of his in-laws, so it was almost second nature to him [1]. This leads us to another troubling exception, which occurs with the bed partner and/or family members who live with the sleepwalker. These individuals are obviously in proximity to the sleepwalker. However, there can be some suspicion of an underlying motive because the sleepwalker typically knows them quite well. In these situations, the relationship between the sleepwalker and the victim should be thoroughly investigated, and any problematic findings should be introduced to the court (as has been the practice thus far).
  Studies of RBD have not been developed as much as those for somnambulism. However, one can still draw several conclusions from anecdotes about episodes of RBD and what is currently known about this disorder. Since this parasomnia involves dream mentation, it differs from somnambulism in terms of the manifestation of intention. Consider the 1858 case of Esther Griggs. In this case, Griggs dreamt that she heard someone screaming “Fire!” She ran into her baby’s bedroom and threw the child out the window to its death in an attempt to save it from the fire in her dream [30]. In this case, an intention is present but only in the parameters of the dream. Other cases often report the defendant dreaming about being attacked [41]. They then try to defend themselves with guns or other objects in the vicinity, hurting people around them in the process. Therefore, in cases of RBD, some haphazard intention within the context of the dream usually does exist, but this intention will rarely make sense out of the dream. Furthermore, the execution of dream intentions would be poor and a little careless – these individuals may utilize any nearby objects to attack whoever is close rather than a selected target. This kind of motivation generally does not qualify as the required *mens rea* for most criminal cases. Compare this scenario to that of delusional schizophrenics. These patients construct a world of illusion, often full of conspiracies against them. As a result, they grow suspicious of particular people around them and may eventually act out against these individuals. When these cases make it to court, these individuals are not considered to have appropriate lucid intention, and they usually qualify for an insanity plea. The intention present in RBD within the dream resembles the intention present in delusional schizophrenics within their delusion. Despite the presence of this sort of distorted motive, it does not fit the necessary standards of *mens rea* and is therefore insufficient for legal accountability.
8. Memory upon waking
  As mentioned earlier, the area in the cerebrum responsible for memory formation and information retention is inactive during sleep. Therefore, any actions that occur during an episode of parasomnia are not remembered [43]. While a defendant with no memory of the event can be frustrating legally, the lack of memory is a significant factor in support of true parasomnias. The amnesia does not have to be complete. Especially in cases of RBD, amnesia would not be akin to a total black out [44]. The presence of dream mentation would result in a fragmented and hazy memory. Still, a significant level of amnesia should be present in order for the defendant to claim that they had been asleep at the time of the violent actions. Of course retrograde amnesia can occur for other reasons, so other potential causes for amnesia should be investigated.
9. Childhood history
  Much debate surrounds the likelihood of children suffering from somnambulism continuing to exhibit parasomnias in adulthood. Because the length of NREM sleep shortens as one grows older, most people actually grow out of it. However, people who sleepwalk during childhood do show higher rates of somnambulism during adulthood than the remainder of the population. This is not a significant factor, as it is statistically unlikely that most children who sleepwalk will continue to do so in adulthood. It can provide some minor support for an argument though. For RBD, this trend does not hold. It has appeared primarily in older populations, so a presence of childhood parasomnia is irrelevant.

## Conclusion

If we take these factors and apply them back to the case of Kenneth Parks, it is possible that a different outcome of the case could occur. Parks was acquitted of both charges. However, at the time of his case, polysomnography was a new diagnostic procedure – its importance was not well understood, and it was not a common test. Therefore, a polysomnography test was not performed and admitted into evidence at Parks’ trial. It should be customary in cases now to conduct a thorough polysomnography evaluation in order to provide additional information for the judge and jury to assess the validity of a plea of parasomnia. As stated earlier, this kind of diagnostic evaluation is the best way to prove a propensity to sleepwalk.

In addition, the contributions of medical practitioners in this case became a sticky situation. This case was one of many that devolved into a mudslinging competition in an attempt to discredit the involved medical opinions. There was also significant suspicion of exaggerated medical opinions in return for incentives provided to the expert witnesses called by each side. If the judge had appointed a non-partisan medical professional, these issues might have been avoided. Some scholars believe that this in itself was the reason the case turned out the way it did, and that an unbiased medical perspective would have led the case in a completely different direction.

Parks’ attorney emphasized Parks’ exacerbating factors: his lack of intention, the accounts of his demeanor at the police station, his stress over his upcoming embezzlement trial, and his childhood history of sleepwalking [1]. Still, many scholars remain quite skeptical about the outcome of the trial. He drove 14 miles to seek out his targets [1]. His relationship with the victims is also of concern, but Parks’ attorneys skillfully addressed these concerns by drawing attention to Parks’ positive relationship with his in-laws and his familiarity with the route to their home [1].

In the end, though it seems easy to imagine that Parks was not sleepwalking, it is difficult to make any assertions as to whether the outcome of the Parks trial should have been different. This demonstrates a clear need for changes in the way violence during episodes of parasomnia are handled in the law and in court. First, the legal system needs a better understanding of parasomnias in order to become familiar with the types of actions possible during these episodes. In addition, a different manner of assigning legal responsibility will improve classification of these actions and prescribe more appropriate legal consequences. This would leave juries with more options (and more appropriate options) than complete acquittal or confinement to a psychiatric hospital. Finally, more specific and detailed guidelines for claiming a plea of parasomnia could resolve some of the heated debate and concern for malingering that typically surrounds these cases. The proposals set forth in this work fill these needs by synthesizing science and law in an attempt to improve the way violent parasomnias are handled.
